# Intestinal Behçet’s disease complicated by myelodysplastic syndrome and secondary pulmonary alveolar proteinosis: a case report

**DOI:** 10.1186/s12876-021-02065-0

**Published:** 2021-12-20

**Authors:** Hiroshi Shimizu, Shuzo Sato, Tomohiro Suzuki, Tomomi Sasajima, Yosuke Takahata, Nobuhiko Shinohara, Kosuke Hideshima, Yuko Yokokawa, Nobuo Matsuhashi, Osamu Ichii, Mayumi Tai, Yutaka Ejiri, Kiori Yano, Takayuki Ikezoe, Hiromasa Ohira, Kiyoshi Migita

**Affiliations:** 1 Department of Gastroenterology, Fukushima Rosai Hospital, Uchigo, Iwaki, Fukushima 973-8403 Japan; 2 Department of Gastroenterology, Fukushima Medical University School of Medicine, Fukushima, Japan; 3 Department of Rheumatology, Fukushima Medical University School of Medicine, 1 Hikarigaoka, Fukushima, Fukushima 960-1295 Japan; 4 Department of Rheumatology, Fukushima Rosai Hospital, Iwaki, Japan; 5 Department of Hematology, Fukushima Medical University School of Medicine, Fukushima, Japan

**Keywords:** Behçet’s disease, Myelodysplastic syndrome, Pulmonary alveolar proteinosis, Trisomy 8, X chromosome anomaly

## Abstract

**Background:**

Gastrointestinal lesions, which sometimes develop in Behçet’s disease (BD), are referred to as intestinal BD. Although rare, intestinal BD can be accompanied by myelodysplastic syndrome (MDS) with abnormal karyotype trisomy 8, which is refractory to immunosuppressive therapy. Pulmonary alveolar proteinosis is a rare lung complication of BD and MDS. Herein, we present an extremely rare case of intestinal BD presenting with MDS and several chromosomal abnormalities, followed by secondary pulmonary proteinosis.

**Case presentation:**

A 58-year-old Japanese woman with a 3-year history of genital ulcers and oral aphthae was admitted to our hospital. The patient developed abdominal pain and persistent diarrhea. Colonoscopy revealed multiple, round, punched-out ulcers from the terminal ileum to the descending colon. Intestinal BD was diagnosed and the patient was treated with colchicine, prednisolone, and adalimumab. However, her symptoms were unstable. Bone marrow examination to investigate the persistent macrocytic anemia revealed the presence of trisomy 8, trisomy 9, and X chromosome abnormalities (48, + 8, + 9, X, i(X) (q10) in 12 out of the examined 20 cells). Based on her hypoplastic bone marrow, the patient was diagnosed with low-risk MDS (refractory anemia). At the age of 61, the patient developed pneumonia with fever and diffuse ground-glass opacities on the lung computed tomography (CT). Chest high-resolution CT and histopathology via transbronchial lung biopsy revealed the presence of pulmonary alveolar proteinosis (PAP). These findings combined with the underlying disease led to the diagnosis of secondary PAP.

**Conclusions:**

Secondary pulmonary proteinosis may accompany intestinal BD with MDS and several chromosomal abnormalities. Physicians should pay attention to lung complications, such as PAP, in patients with intestinal BD complicated by MDS. Genetic abnormalities may be associated with the development of such diseases.

## Background

Behçet’s disease (BD) is a systemic inflammatory disorder of unknown etiology and is characterized by oral ulcers, genital ulcers, uveitis, skin lesions, and arthritis. Lesions in the gastrointestinal tract, central nervous system, and blood vessels sometimes accompany BD [1]. Gastrointestinal involvement in BD, called intestinal BD, results in higher morbidity and mortality [2]. Myelodysplastic syndrome (MDS) with trisomy 8, a hematological disorder, may also be associated with intestinal BD [3–6]. Intestinal BD associated with trisomy-8-positive MDS is refractory to various immunosuppressive treatments, including tumor necrosis factor (TNF) inhibitors, and some patients require hematopoietic stem cell transplantation to achieve complete remission [5–7].

Pulmonary alveolar proteinosis (PAP) is a rare disorder with unknown etiology and is characterized by the accumulation of phospholipids and surfactant proteins in the alveolar lumen and terminal bronchiole [8–10]. PAP is classified into autoimmune, secondary, and congenital PAP [11]. In autoimmune PAP, the granulocyte/macrophage colony-stimulating factor (GM-CSF) cascade is disrupted by high levels of GM-CSF antibody in the lungs [12]. Secondary PAP (SPAP) accompanies underlying diseases; surfactant clearance is impaired by abnormal numbers and functions of alveolar macrophages in secondary PAP [9, 11, 13]. MDS is a major underlying condition that leads to SPAP [8, 11]. Lung complications in BD patients are uncommon and intestinal BD with MDS complicated by PAP is particularly rare [8]. Here, we present an extremely rare case of intestinal BD with MDS. The patient shows several other chromosomal abnormalities, including trisomy 8, trisomy 9, and altered X chromosome, complicated by SPAP.

## Case presentation

**Fig. 1:**
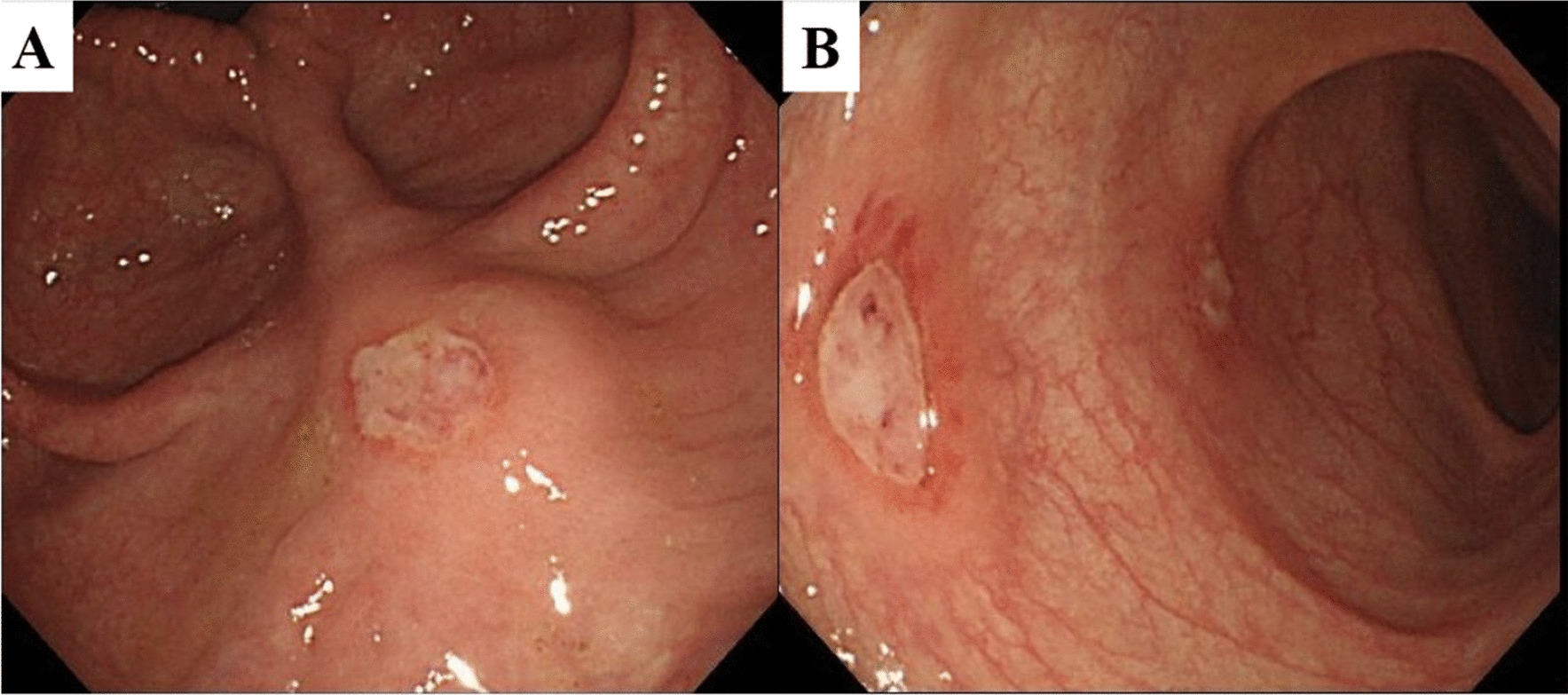
Colonoscopy findings of intestinal Behçet’s disease on admission. (**a**, **b**) Colonoscopy showed round, punched-out ulcers in the ileocecal region

**Fig. 2:**
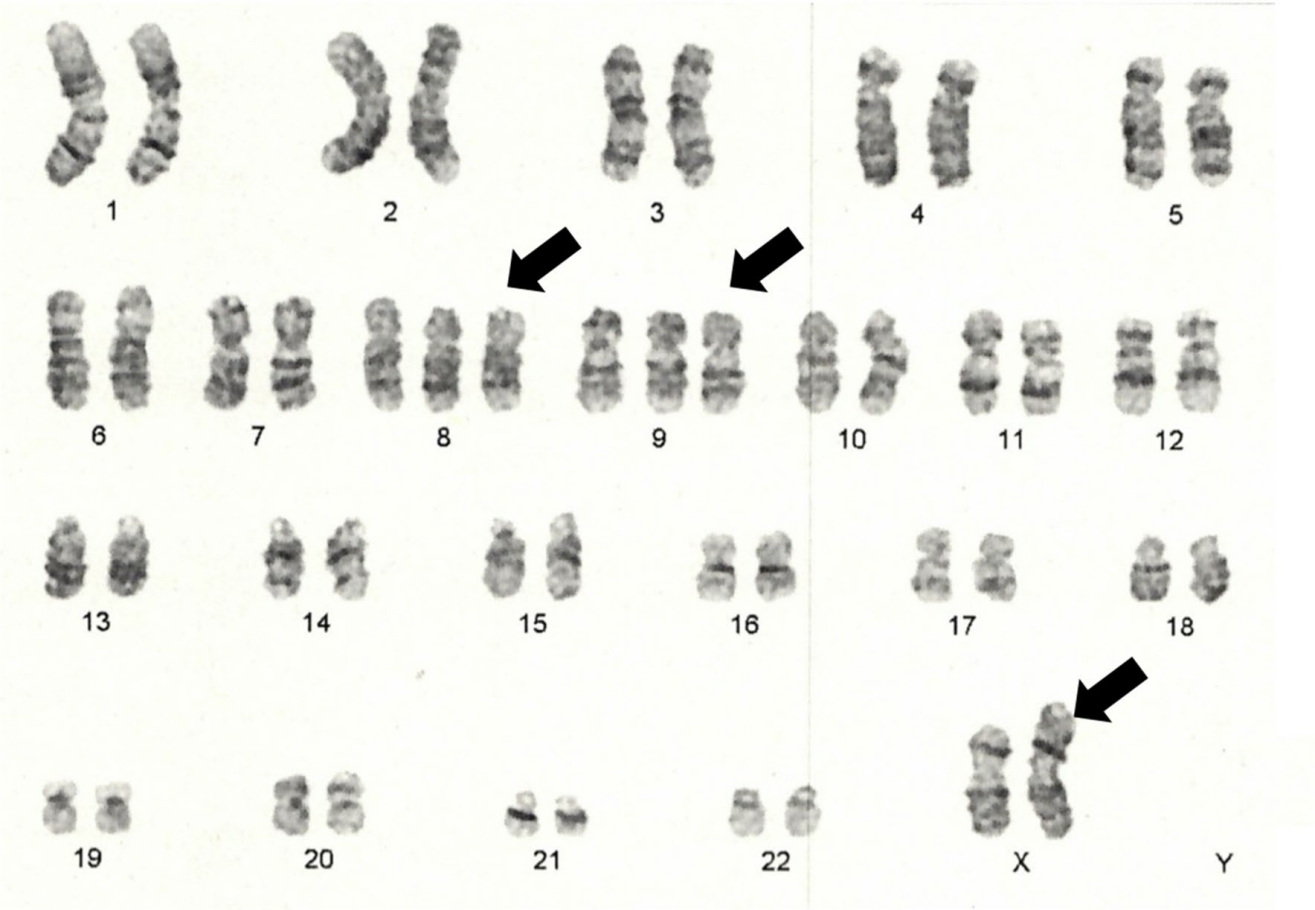
Chromosome examination of bone marrow cells. Chromosome examination revealed the presence of trisomy 8, trisomy 9, and X chromosome abnormalities (48, X, i(X)(q10), + 8, + 9). Abnormal chromosomes are indicated by arrows

**Fig. 3:**
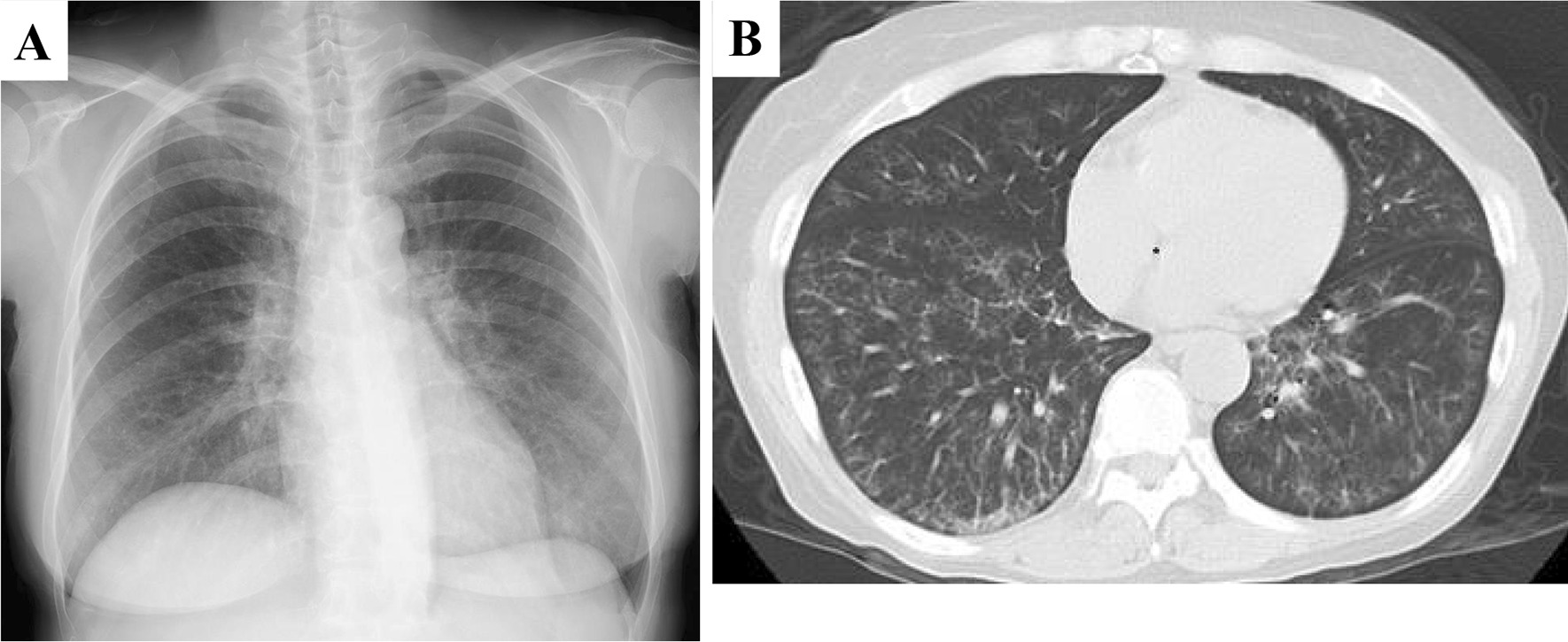
Chest X-ray and computed tomography of the lung. **a** Chest X-ray showed ground-glass opacification mainly in both lower lungs. **b** Chest computed tomography showed widely distributed ground-glass opacification of the alveolar spaces in both lungs

**Fig. 4:**
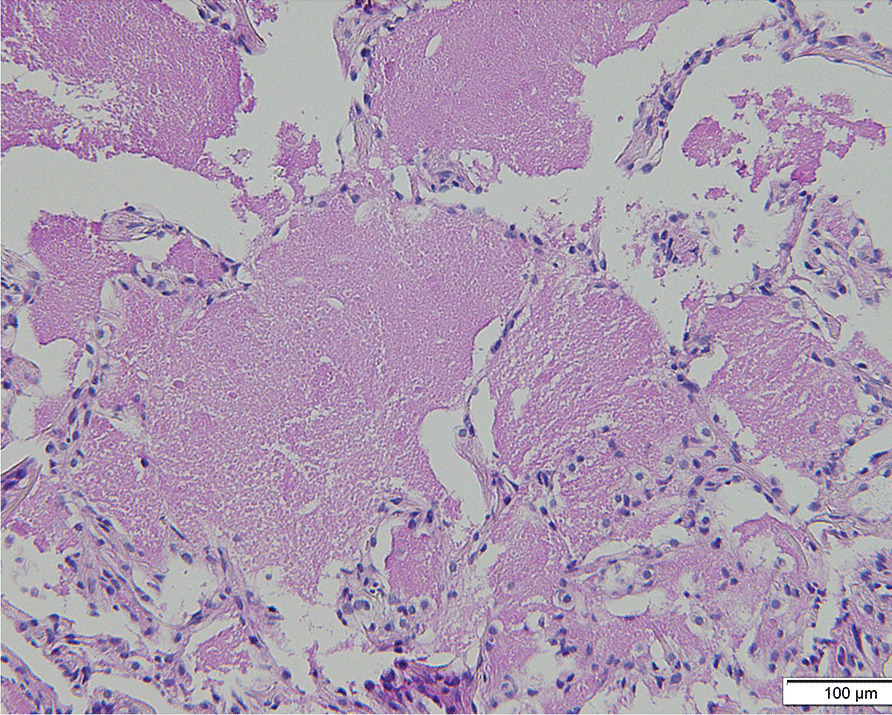
Histopathology with periodic acid–Schiff staining obtained by transbronchial lung biopsy. The slide showed alveoli filled with an amorphous and acellular eosinophilic material, indicating the presence of pulmonary alveolar proteinosis (magnification, 200 ×)

A 58-year-old Japanese woman with a 3-year history of genital ulcers and oral aphthae was admitted to our hospital. She had a history of uterine fibroids and thrombocytopenia during pregnancy. Her family history included hypertension and diabetes in her mother and MDS in her offspring. A year ago on admission, she developed abdominal pain and persistent diarrhea. Colonoscopy revealed multiple colonic ulcers, and she was referred to our hospital. On physical examination, we found erythema nodosum without uveitis on the left forearm. Laboratory tests revealed macrocytic anemia (red blood cell count, 251 × 10^4^/μl; hemoglobin level, 9.3 g/dl). White blood cell and platelet counts were 4800/μl and 14.3 × 10^4^/μl, respectively. Serum C reactive protein levels were 0.35 mg/dl and anti-nuclear antibody was negative. Human leukocyte antigen analysis was positive for B51 and A26. Colonoscopy showed multiple, round, punched-out ulcers from the terminal ileum to the descending colon (Fig. 1a, b). Intestinal Behçet’s disease (BD) was diagnosed, and she received 3600 mg of mesalazine, 0.5 mg of colchicine, and 30 mg of oral prednisolone per day. Adalimumab, a TNF inhibitor was also added for maintenance therapy. However, during steroid tapering, her abdominal symptoms relapsed. Persistent anemia was observed and bone marrow examination was performed. The results revealed the presence of trisomy 8, trisomy 9, and X chromosome abnormalities (48, + 8, + 9, X, i(X) (q10) in 12 out of the 20 cells examined; Fig. 2). The patient’s bone marrow was hypoplastic with the appearance of micromegakaryocytes and < 1% of atypical cells, resulting in the diagnosis of low-risk MDS (refractory anemia). At the age of 60, Infliximab (5 mg/kg) against refractory intestinal BD was initiated instead of Adalimumab. Infliximab was temporary effective for abdominal symptoms, however, she developed pneumonia with fever at the age of 61. Chest X-ray and lung computed tomography (CT) showed diffuse ground-glass opacities in both lungs (Fig. 3a, b). High-resolution chest CT and histopathology via transbronchial lung biopsy revealed the presence of pulmonary alveolar proteinosis (Fig. 4). Her serum GM-CSF concentration was 4.3 pg/ml (normal range, < 5 pg/ml) and an anti-GM-CSF antibody was negative. Based on findings with the underlying disease, a diagnosis of SPAP was established. She was treated with infliximab (5 mg/kg) for active intestinal BD for every 4 weeks and received whole lung lavage to improve respiratory symptoms with SPAP. She is now preparing to receive bone marrow transplantation as a curative treatment.

## Discussion and conclusions

We present a case of intestinal BD accompanying by MDS with several chromosomal abnormalities, including trisomy 8. The patient’s symptoms were refractory to immunosuppressive therapy and complicated by PAP without anti-GM-CSF antibodies, indicating SPAP. To the best of our knowledge, this is the first case of intestinal BD associated with trisomy-8-positive MDS accompanied by SPAP and several chromosomal abnormalities, including chromosome X. Cases of intestinal BD with trisomy-8-positive MDS complicated by SPAP are extremely rare; only two such cases have been described in the literature (Table 1) [8]. Including our case, two of the three cases were female. Past cases showed refractory anemia in MDS type and no eye lesions of BD symptoms were found. Our patient only had HLA-B51, trisomy 9, and X chromosome anomaly. Chest CT showed diffuse ground-glass opacities in both lungs in all patients.

**Table 1:** Summary of intestinal Behçet’s disease patients with myelodysplastic syndrome associated with secondary pulmonary alveolar proteinosis

Author/year (reference)	Age/sex	MDS type	BD symptoms	Chromosomal abnormalities/HLA	Respiratory symptoms	HRCT findings (GGO pattern)	Treatment before MDS onset (duration, years)	Outcome
Handa/2014 [8]	49/F	RA	G, I, O, S	Trisomy 8/HLA-B51-	None	Diffuse	Cyclosporine A, Prednisolone, Sulfasalazine, TNF-inhibitor (14 years)	Dead
Handa/2014 [8]	33/M	RA	I, O, S	Trisomy 8/HLA-B51 -	Cough	Diffuse	Azathioprine, Prednisolone, Sulfasalazine, TNF inhibitor (5 years)	Dead
Present case/2021	58/F	RA	G, I, O, S	Trisomy 8, Trisomy 9, X, i(X)(q10)/HLA-B51+	Cough Fever	Diffuse	Colchicine, Methalazine, Prednisolone, TNF inhibitor (3 years)	alive

BD, Behçet’s disease; G, genital ulcer; GGO, ground-glass opacity; HLA, human leukocyte antigen; HRCT, high-resolution computed tomography; I, intestinal lesions; MDS, myelodysplastic syndrome; O, oral ulcer; RA, Refractory anemia; S, skin lesion; TNF, tumor necrosis factor

Despite treatment with steroids and immunosuppressants, including TNF inhibitors, the prognosis of such cases remained poor because of severe infection and in part, deterioration of PAP [8]. Indeed, the treatment of SPAP in patients with MDS is challenging [11, 14]. Whole lung lavage can be considered, but the effect of this treatment is temporary [10]. Zhang et al. have reported that patients with SPAP secondary to hematological diseases had poor survival compared with those with other causes, partly due to infections [15]. Conversely, a previous report has described successful treatment by bone marrow transplantation for patients with SPAP and MDS [16]. Bone marrow transplantation can be considered in these cases but is not applicable for all patients. In Japan, Ishii et al. reported that patients with MDS and SPAP were treated with bone marrow transplantation; however, three of seven patients died from pneumonia within 3 months of transplantation [11]. In another report, patients with intestinal BD and trisomy-8-positive MDS who were refractory to immunosuppressive therapy received bone marrow transplantation as a cure for both diseases [6, 7]. Transplantation was successful in some patients, but severe infections were a major issue preventing recovery [6]. Further accumulation of such cases is needed to establish an appropriate treatment. The distinct mechanism underlying the development of intestinal BD associated with MDS and SPAP is unclear, however, several genetic abnormalities may be involved in the pathogenesis.

Trisomy 8 was postulated to be involved in the inflammatory processes of intestinal BD through the production of abnormal inflammatory cytokines, such as GM-CSF, interleukin (IL)-1beta, IL-6, IL-8, IL-17, IL-18, TNF-alpha, and interferon-gamma [17, 18]. The upregulation of inflammatory genes was detected in CD34-positive progenitor cells in patients with MDS and trisomy 8 [19]. Inflammation may influence the production of abnormal macrophages in the lungs, resulting in PAP. Furthermore, Moriyama et al. have reported that alveolar cells obtained from bronchoalveolar lavage had trisomy 8, indicating alveolar macrophages were likely differentiated from abnormal hematopoietic stem cells with trisomy 8 [9]. This hypothesis is supported by the fact that hematopoietic stem cell transplantation has alleviated MDS with SPAP patients [16]. In contrast, trisomy 9 is rarely reported and is chiefly associated with myeloproliferative and myelodysplastic disorders, such as MDS [20]. Disrupted GM-CSF signaling is a major cause of PAP. GM-CSF regulates alveolar surfactant homeostasis. Moreover, genetic abnormalities of the GM-CSF receptor α-chain, encoded by the X-chromosome, can cause PAP [21]. The X chromosome abnormalities might have contributed to the development of PAP by affecting GM-CSF signaling even in the absence of anti-GM-CSF autoantibodies in this case. Taken together, these genetic abnormalities can synergistically affect each other, altering organ environments including inflammatory cytokine expressions such as GM-CSF, abnormal cell proliferation and finally resulted in the manifestation of SPAP in intestinal BD with MDS [9, 16–18].

In conclusion, intestinal BD with MDS may be accompanied by lung complications in rare instances. Thus, attention should be paid to the development of PAP as a lung complication in patients with intestinal BD with trisomy-8 positive MDS. Genetic and hematological investigations are important in establishing an early diagnosis in such cases. Several chromosomal abnormalities, including trisomy 8 and X chromosome anomaly, may contribute to the development of PAP in combination with BD symptoms, which impacts patient outcomes.

## Data Availability

Data sharing is not applicable to this article because no datasets were generated or analyzed during the current study.

## Abbreviations

BD: Behçet’s disease
CD: Cluster of differentiation
CT: Computed tomography
GM-CSF: Granulocyte/macrophage colony-stimulation factor
GM-CSFR: Granulocyte/macrophage colony-stimulation factor receptor
HSCT: Hematopoietic stem cell transplantation
IL: Interleukin
MDS: Myelodysplastic syndrome
PAP: Pulmonary alveolar proteinosis
SPAP: Secondary pulmonary alveolar proteinosis
TNF: Tumor necrosis factor
